# Curcumin-Loaded Solid Lipid Nanoparticles Enhanced Anticancer Efficiency in Breast Cancer

**DOI:** 10.3390/molecules23071578

**Published:** 2018-06-29

**Authors:** Wenrui Wang, Tiantian Chen, Henan Xu, Baihui Ren, Xiaodan Cheng, Rongrong Qi, Haibo Liu, Yueyue Wang, Lei Yan, Sulian Chen, Qingling Yang, Changjie Chen

**Affiliations:** 1 Department of Biotechnology, Bengbu Medical College, Bengbu 233030, China; wenrui-wang1983@163.com (W.W.); 18895689061@163.com (B.R.); 18895650868@163.com (X.C.); r18895640760@163.com (R.Q.); 2 AnHui Province Key Laboratory of Translational Cancer Research, Bengbu Medical College, Bengbu 233030, China; ttctoyy@gmail.com (T.C.); z0170615@163.com (H.X.); 18755295203@163.com (Y.W.); 13145520690@sina.cn (L.Y.); 3 Department of Public Foundation, Bengbu Medical College, Bengbu 233030, China; haibo_liu1997@163.com; 4 Department of Biochemistry and Molecular Biology, Bengbu Medical College, Bengbu 233030, China; csl9557@sina.com

**Keywords:** solid lipid nanoparticle, curcumin, breast cancer, cyclin D1

## Abstract

Curcumin (Cur) has been widely used in medicine, due to its antibacterial, anti-inflammatory, antioxidant, and antitumor effects. However, its clinic application is limited by its instability and poor solubility. In the present wok, curcumin was loaded into solid lipid nanoparticles (SLNs), in order to improve the therapeutic efficacy for breast cancer. The results measured using transmission electron microscopy (TEM) indicated that Cur-SLNs have a well-defined spherical shape; the size was about 40 nm with a negative surface charge. The drug loading and encapsulation efficiency in SLNs reached 23.38% and 72.47%, respectively. The Cur-SLNs showed a stronger cytotoxicity against SKBR3 cells. In vitro cellular uptake study demonstrated a high uptake efficiency of the Cur-SLNs by SKBR3 cells. Moreover, Cur-SLNs induced higher apoptosis in SKBR3 cells, compared to cells treated by free drug. In addition, Western blot analysis revealed that Cur-SLNs could promote the ratio of *Bax/Bcl-2*, but decreased the expression of *cyclin D1* and *CDK4*. These results suggested that Cur-SLNs could be a potential useful chemotherapeutic formulation for breast cancer therapy.

## 1. Introduction

Breast cancer is one of the most common cancers in women in the world [1]. The rate of breast cancer incidence is increasing rapidly because of the changes in multiple environmental, hormonal, and lifestyle risk factors [2,3,4]. Many kinds of therapy, such as chemotherapy and radiotherapy, have been tried for treatment of breast cancer. However, these therapies were often accompanied by many side effects [5].

Curcumin (Cur) is a hydrophobic polyphenol, derived from the plant curcuma longa (turmeric), with low intrinsic toxicity. It has been reported to possess a variety of pharmacologic effects, including antibacterial, anti-inflammatory, antioxidant, and antitumor properties [6,7,8]. However, curcumin is highly hydrophobic: the instability and poor bioavailability are major drawbacks for its further clinical application [9,10,11]. Therefore, there is need for finding new strategies to improve the physicochemical properties and therapeutic efficacy of curcumin.

Recent development of drug delivery systems, such as nanocarriers, is gaining increasing attention, due to its ability to improve the anticancer properties of various small molecules. During the past few years, solid lipid nanoparticles (SLNs) have attracted much attention in the field of drug delivery. SLNs present some excellent material properties, such as small particle size, biocompatibility, chemical and mechanical stability, and easy functionalization ability [12,13]. In particular, the physiological lipid core within SLNs can protect the encapsulated drugs from chemical degradation and enhance their physical stability. In addition, SLNs have been reported to modulate release kinetics, improve blood circulation time, and increase overall therapeutic efficacy of anticancer drugs [14,15].

In the current study, we primarily aimed to prepare SLNs to effectively deliver curcumin to treat breast cancer. For this purpose, curcumin-loaded solid lipid nanoparticles (Cur-SLNs) were prepared and characterized in terms of morphology, particle size and zeta potential. The anticancer effect of free curcumin and Cur-SLNs was investigated in SKBR3 cancer cells. The cellular uptake ability was also evaluated. Furthermore, mechanism of cytotoxicity Cur-SLNs against human breast cancer cells was assessed. This study indicates that Cur-SLNs could be a potential useful chemotherapeutic formulation for breast cancer therapy.

## 2. Results

### 2.1. Characterization of Cur-SLNs

**Figure 1:**
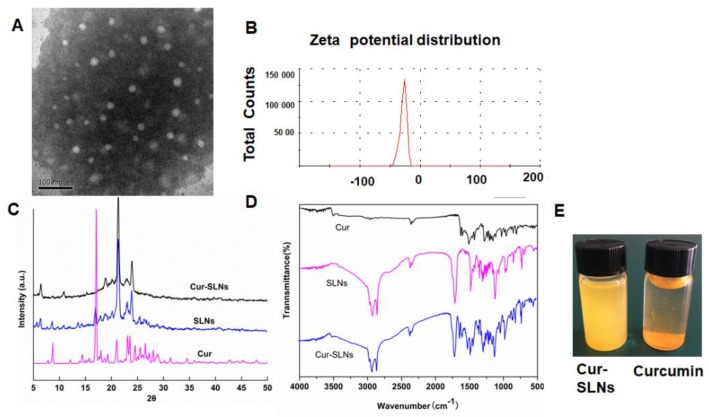
(**A**) Transmission electron microscopy (TEM) of curcumin-loaded solid lipid nanoparticles (Cur-SLNs), scale bar, 100 nm; (**B**) The zeta potential of Cur-SLNs; (**C**) X-ray diffraction (XRD) curves of SLNs, curcumin, and Cur-SLNs; (**D**) FTIR analysis of SLNs, curcumin, and Cur-SLNs; (**E**) Solubility of free Cur and Cur-SLNs in PBS solution.

Transmission electron microscopy (TEM) studies were carried out to evaluate the morphology of the Cur-SLNs. As can be seen in Figure 1A, the Cur-SLNs particles were spherical, with smooth morphology. Most of the particles were observed to be distributed between 30 and 50 nm under TEM. Zeta potential is a significant factor to maintain the stability of nanoparticles in suspension through the electrostatic repulsion between particles [16]. As shown in Figure 1B, the zeta potential value of Cur-SLNs was about −25.3 ± 1.3 mV, which was high enough to make the nanoparticles repel each other, thereby avoiding particle aggregation and keeping the long-term stability of nanoparticles. The drug-loading and encapsulation efficiencies of curcumin by SLNs were 23.38% and 72.47%, respectively.

X-ray diffraction (XRD) method was used to clarify the existing form of curcumin after encapsulation into SLNs. The diffraction patterns of the curcumin, SLNs, and Cur-SLNs are shown in Figure 1C. The pure curcumin exhibits sharp peaks in the range of 10–30°, which suggests a high crystalline structure [17], but these characteristics are not apparent in the Cur-SLNs, which indicates that curcumin entrapped in the lipid core of SLNs was in the amorphous or disordered-crystalline phase. In addition, SLNs exhibit similar diffraction patterns with Cur-SLNs, which suggests that the encapsulation of curcumin did not change the nature of the SLNs. In Fourier transform infrared spectroscopy analysis (FTIR) spectrum of curcumin, Cur-SLNs, and SLNs was shown in Figure 1D, in which curcumin showed a number of characteristic bands [18]. Among these, the absorption peak at 1627 cm^−1^ could correspond to C=C and C=O stretching, 1509 cm^−1^ assigned to C=O, and the absorption at 1281 cm^−1^ assigned to C–O stretching. The FTIR spectra for SLNs and Cur-SLNs did not have any peak shift or loss of functional groups, suggesting that curcumin was compatible with other ingredients used in the preparation of SLNs formulation.

As we known, poor aqueous solubility has restricted the clinical applications of Cur. Therefore, to confirm the nano-formation was able to improve its solubility, equal amounts of free Cur and Cur-SLNs were suspended in an equal volume of PBS solution (pH 7.4). As shown in Figure 1E, it was observed that free Cur is hardly soluble in aqueous media with visible precipitation. By contrast, Cur-SLNs could be dispersed homogeneously in aqueous solution.

### 2.2. In Vitro Cytotoxic Activity and Cellular Uptake Study

**Figure 2:**
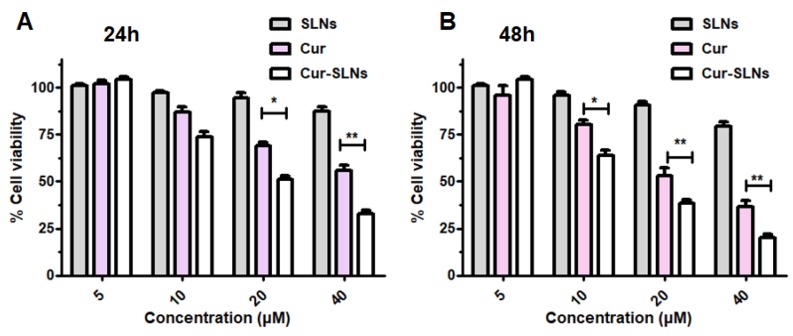
In vitro cell viability analysis of SLNs, Cur, and Cur-SLNs in SKBR3 breast cancer cells. The cells were treated with the respective formulations, and incubated for (**A**) 24 h and (**B**) 48 h. Data are represented as percentage of viable cells. * *p* < 0.05; ** *p* < 0.01.

The in vitro cytotoxic activity of curcumin, and Cur-SLNs on SKBR3 cancer cells was investigated by sulforhodamine B (SRB) assay. As shown in Figure 2, free Cur and Cur-SLNs inhibited the cell proliferation in a time- and dose-dependent manner, whereas there were no significant cytotoxic effects of free SLNs on cell viability. The IC50 value was used to evaluate the cytotoxic effect of Cur-SLNs. After 48 h incubation, the IC50 value was 28.42 μM and 18.78 μM for free Cur and Cur-SLNs, respectively. These results suggested that the use of SLNs improve the ability of curcumin to inhibit cell proliferation in vitro.

**Figure 3:**
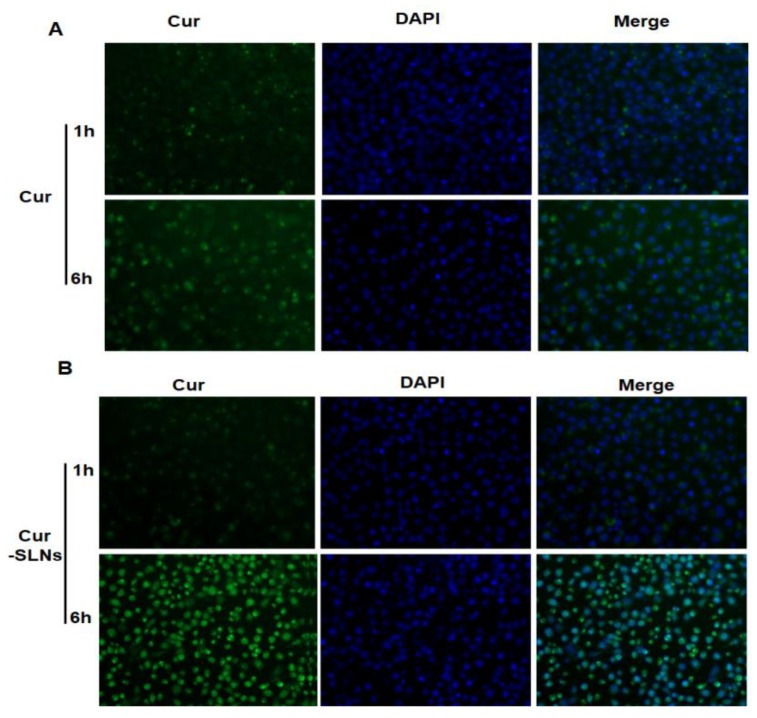
Representative fluorescent images from the (**A**) curcumin and (**B**) Cur-SLNs internalization in SKBR3 cancer cell line at 1 h and 6 h. Left panel refers to internalized curcumin, that shows green fluorescence color; middle panel is nuclei stained by 4′,6-diamidino-2-phenylindole (DAPI), thus exhibiting blue regions; right panel is an overlay image of both DAPI and curcumin.

In order to check whether the increased cytotoxicity of Cur-SLNs is due to higher uptake of curcumin in the form of nanoparticles, cellular uptake study was investigated. SKBR3 cells were treated with free curcumin and Cur-SLNs for 6 h. The cells were stained with 4’ -diamidino-2-phenylindole (DAPI) that was a DNA-selective probe excited by ultraviolet light and showed strong blue fluorescence when binding to DNA. Therefore, the cells incubated with curcumin exhibited either green (due to curcumin) or blue (due to DAPI). As shown in Figure 3, the fluorescence intensity of Cur-SLNs treated cells were higher than free Cur treated at the same time, these results indicated that Cur-SLNs had a higher cellular uptake ability than free Cur.

### 2.3. Effects of Cur-SLNs on Reactive Oxygen Species (ROS) Production

**Figure 4:**
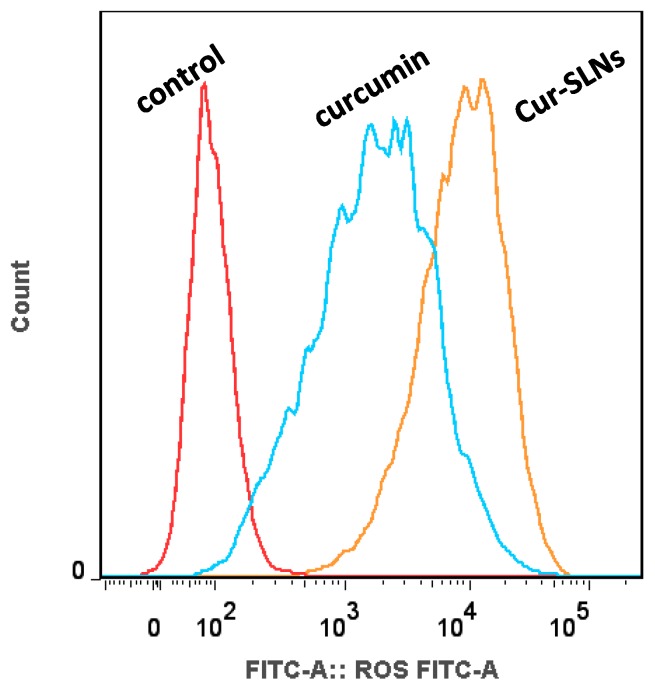
Dichlorodihydrofluorescein diacetate (DCFH-DA)/DCFH assay performed for the detection of Reactive Oxygen Species (ROS) generation by Cur and Cur-SLNs in SKBR3 cells.

Next, we used 2′,7′-dichlorodihydrofluorescein diacetate (DCFH-DA) as a fluorescent probe to measure the change of ROS. The results demonstrated that after treatment with free curcumin and Cur-SLNs, the generation of ROS in SKBR3 cells was significantly changed. We observed an increase in ROS levels following treatment of cells with both formulations for a certain period of time (Figure 4). Compared with free curcumin, the augmentation of ROS levels treated with Cur-SLNs was significantly enhanced in SKBR3 cells.

### 2.4. Effects of Cur-SLNs on Apoptosis Induction and Cell Cycle

**Figure 5:**
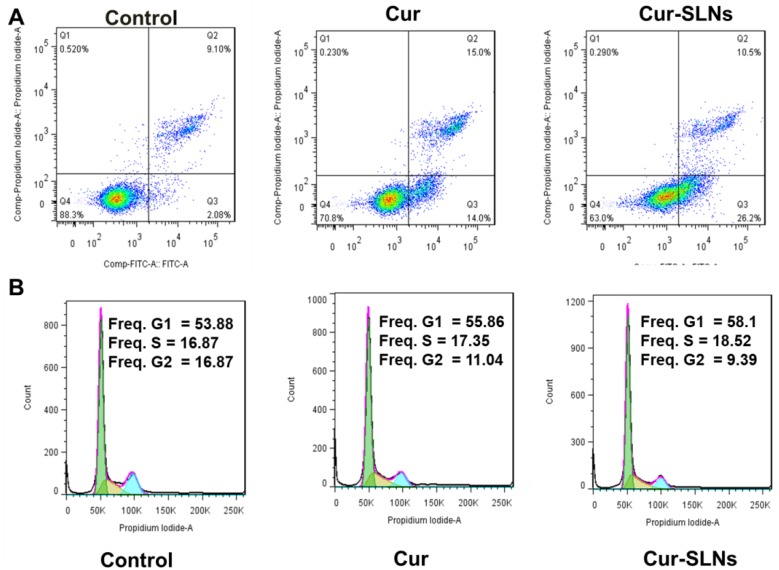
(**A**) Apoptosis and (**B**) cell cycle were analyzed in SKBR3 cells after 24 h treatment with curcumin and Cur-SLNs.

Furthermore, fluoresceine isothiocyanate (FITC)-conjugated Annexin V and propidium iodide (PI) were used to measure the numbers of apoptotic and necrotic cells [19]. As seen in Figure 5A, when cells were treated with free curcumin, the apoptosis rates were gradually increased from 11.18% to 29%. As expected, a significantly higher fraction of apoptosis was observed in Cur-SLN-treated cells (36.7%) than free curcumin.

To further identify the mechanism behind the cell growth inhibition and apoptosis, the effect of free curcumin and Cur-SLNs on the cell cycle of SKBR3 cancer cells was evaluated. As depicted in Figure 5B, free Cur and Cur-SLNs treatment cause the G1/S and G2/M checkpoints’ activation in SKBR3 cells. Cur-SLNs treatment led to a much higher proportion of cells in G1 than curcumin alone. The results revealed that the Cur-SLNs could induce cell cycle arrest at G1/S phase. These findings were consistent with the results of in vitro cytotoxicity.

### 2.5. Effects of Cur-SLNs on Cell Migration

**Figure 6:**
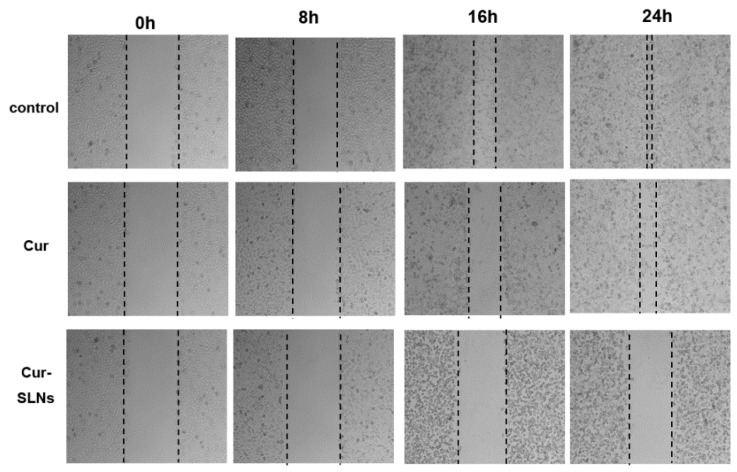
Wound-healing assays were performed to assess cell migration. SKBR3 cells were seeded in migration chambers and treated with curcumin and Cur-SLNs at 20 μM. Images of the migration area were photographed 0 h, 8 h, 16 h, and 24 h. Representative images taken from duplicated experiments.

In order to further examine the possible anti-migration ability of curcumin and Cur-SLNs, a wound-healing assay was performed. Figure 6 shows the representative images of a scraping assay after treatment with free curcumin and Cur-SLNs at different times. The results of wound-healing assay demonstrated both formulations decreased cell migration in a time-dependent manner. The migration of SKBR3 cells was significantly inhibited in the presence of the free Cur and Cur-SLNs, and the Cur-SLN treatment showed more pronounced effects on cell motility.

### 2.6. Western Blot Analysis

**Figure 7:**
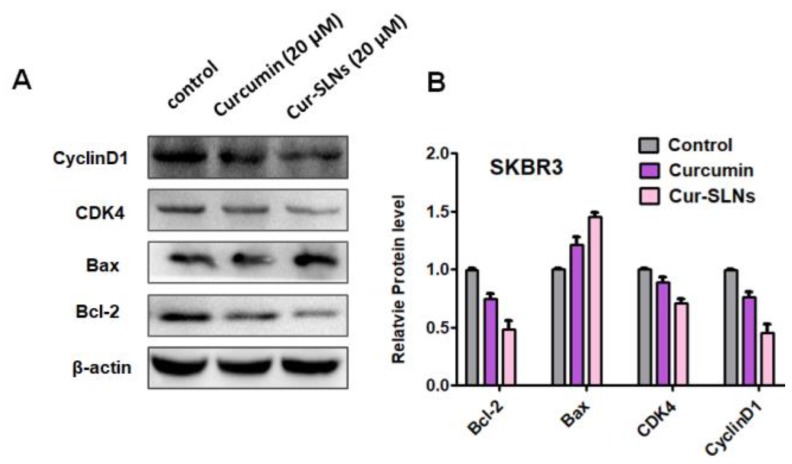
(**A**) The protein expression levels of *Bcl-2, Bax, CDK4*, and *cyclin D1*. *β-Actin* was used as a loading control. (**B**) Quantification of levels of the abovementioned proteins, normalized to the internal control (*n* = 3 per group, mean ± SD).

In order to further to understand the mechanism for the anticancer effects of Cur and Cur-SLNs, several key apoptosis-associated proteins were assessed via Western blot analysis (Figure 7). As expected, the expression level of pro-apoptotic protein *Bax* was increased while that of anti-apoptotic protein *Bcl-2* was decreased in SKBR3 cells after 48 h treatment. Moreover, molecular determinant of cell cycle was studied, and the results showed that Cur-SLNs were more effective than Cur at decreasing the expression of *cyclin D1* and *CDK4*. This result indicates that Cur-SLNs could arrest cell cycle at G1/S. Taken together, Cur-SLNs could more greatly induce apoptosis and cell cycle arrest.

## 3. Discussions

Breast cancer is one of the greatest threats to women’s health, globally. Currently, chemotherapy is the principle strategy for cancer treatment, but it has various toxic side effects. Nanomedicine for cancer treatment has aroused great and extensive interest nowadays [20,21,22].

Previous studies have demonstrated that curcumin has anticancer potential, by regulating cell cycle, apoptosis and survival, proliferation, invasion, and metastasis. However, as a natural compound, its clinical application is limited by the low bioavailability and fast elimination. Yallapu et al. had reported that the curcumin-loaded cellulose nanoparticles showed improved anticancer efficacy compared to free curcumin [23]. In this study, SLNs has been applied to enhance the water solubility and bioavailability of curcumin. First, Cur-SLNs were fabricated by the emulsification evaporation-low temperature solidification method. The solid lipid nanoparticles were developed using biocompatible and biodegradable materials.

The surface charge and size of nanoparticles are important parameters affecting the cellular damage of cancer cells [24]. The resultant uniform sizes of the Cur-SLNs enable better solubility and bioavailability. The zeta potential was negative enough to sustain the stability of Cur-SLNs dispersed system. FTIR and XRD analyses have revealed that there were no drug–lipid interactions in the curcumin-encapsulated SLNs, which is necessary to identify the drug–lipid interactions, as it may affect the entrapment efficiency and stability of the SLNs.

In the present study, we checked the effect of curcumin and Cur-SLNs on the cell proliferation of SKBR3 cells by SRB assay. Our data indicated that the Cur and Cur-SLNs inhibited cell proliferation in a dose-dependent manner, and the IC50 value of curcumin and Cur-SLNs on SKBR3 cells is 28.42 µM and 18.78 µM, respectively. The results demonstrated that the Cur-SLNs had highly cytotoxic effects on SKBR3 cells. This can be attributed to the improved cellular uptake when the cells are treated with Cur-SLNs. 

Cellular uptake potential of the nanoparticles system is very important from a therapeutic efficacy perspective. The enhancement of cellular uptake of the drug formulation was also studied. After 1 h of incubation, an obvious green fluorescence could be observed in SKBR3 cells treated with Cur-SLNs, indicating the successful cellular internalization of the Cur-SLNs. The fluorescence intensity became stronger after 6 h of incubation in SKBR3 cells, while free Cur showed only weak green fluorescence. The enhanced cytotoxicity of Cur-SLNs observed by SKBR3 cells compared to curcumin in the SRB assay is due to the efficient uptake of nanoparticles. It appears that free curcumin enters into the cell by passive diffusion, and Cur-SLNs were constantly uptaken by cells possibly by an energy-dependent transport pathway [5,25].

ROS burst and mitochondrial membrane potential disruptions are significant markers of oxidative stress and initiators of cellular apoptosis [26]. Recently, curcumin has been reported to induce apoptosis by elevating ROS generation [27]. In this study, after treatment with free curcumin and Cur-SLNs, the generation of ROS in SKBR3 cells was significantly enhanced, suggesting that the elevated ROS might contribute to the enhanced cell apoptosis. It has also been shown that high levels of ROS can damage the mitochondrial membrane, causing it to become depolarized [28]. Depolarization of the mitochondrial membrane can lead to destabilization of *Bcl-2*. The ratio of *Bcl-2/Bax* expression was quantified via Western blot in SKBR3 cells after treatment with either curcumin or Cur-SLNs compared to untreated cells. In line with the previous results, the Cur-SLNs significantly increased their production of ROS, and dramatically decreased the ratio of *Bcl-2/Bax* compared to free curcumin.

Furthermore, to identify the mechanism behind the cell growth inhibition and apoptosis, cell cycle progression was studied. In our study, the results showed that Cur and Cur-SLNs induced cell cycle arrest at G2/M phase. The results are in agreement with previous studies that nanocurcumin inhibited cell proliferation by inducing G1/S phase arrest in Caski and SiHa cells [29].

It is well known that cyclins are the proteins which control cell cycle progression. *Cyclin D1* is a product of the *CCND1* gene [30], which is considered a well-established human oncogene [31]. The gene and its product have been extensively examined in cancer and there are many studies that confirmed their involvement in breast, lung, colon, bladder, and liver cancers. *CDK4* activity is deregulated in many human tumors [5]. *CDK4* has been shown to be absolutely crucial for various oncogenic transformation processes, suggesting that many cancer cells may be addicted to high *CDK4* activity [32].

**Figure 8:**
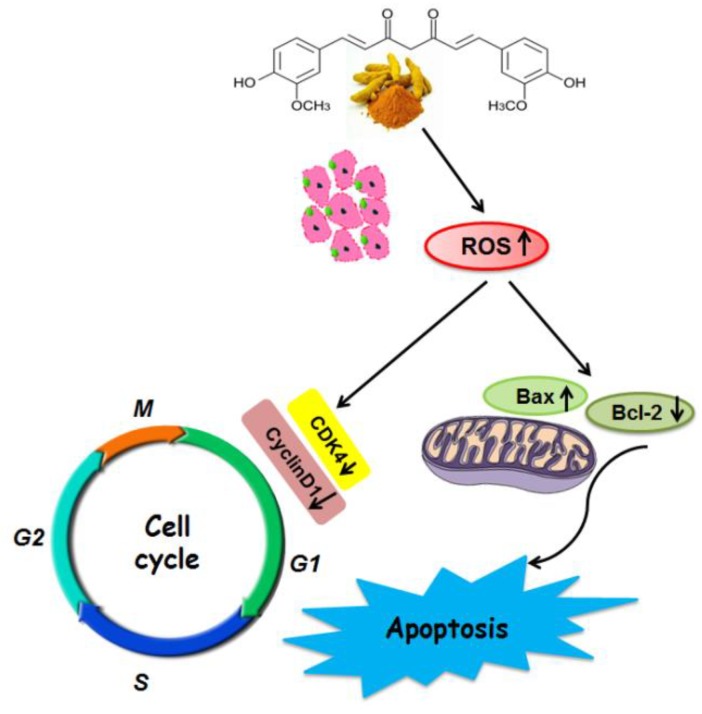
Schematic diagram shows the mechanisms underlying the inhibition of SKBR3 cells by curcumin and Cur-SLNs.

*Cyclin D1/CDK4* and *Cyclin E/CDK2* regulate transition in the G1 phase, while *cyclin A/CDK2* regulates the entry into G2/M phase [33]. In the present study, curcumin decreased the level of *Cyclin D1* and *CDK4*. Cur-SLNs have a profound inhibitory effect on the level of cell cycle-related proteins (Figure 8).

## 4. Materials and Methods

### 4.1. Reagents

Curcumin was purchased from Aladdin Chemicals (Shanghai, China). Stearic acid, lecithin chloroform, and Tween^®^80 were obtained from Sinopharm Chemical Reagent Co, Ltd. (Shanghai, China). DMEM (Dulbecco’s Modified Eagle Medium), and fetal bovine serum (FBS), penicillin G, trypsin-EDTA, 4′,6-diamidino-2-phenylindole(DAPI), and 2′,7′-dichlorodihydrofluorescein diacetate (DCFH-DA) were obtained from KeyGen Biotech Co (Nanjing, China). Other chemicals used in this study were of analytical grade. The primary antibodies against Bcl-2, Bax, CDK4, and CyclinD1 were purchased from Proteintech (Beverly, MA, USA). Horseradish peroxidase (HRP)-conjugated secondary antibodies against rabbit or mouse immunoglobulin were purchased from Santa Cruz Biotechnology (Santa Cruz, CA, USA).

### 4.2. Preparation of Nanoparticles

Cur-SLNs were prepared by the emulsification and low-temperature solidification method. Briefly, 0.15 g of curcumin, 0.2 g of stearic acid, and 0.1 g of lecithin were dissolved in 10 mL of chloroform, by ultrasound, to form the organic phase. The aqueous phase was composed of 0.2 g of Myrj52 dissolved in 30 mL of distilled water. Then, the organic phase was added into the aqueous phase, and the mixture was stirred at 1000 rpm at 75 °C for approximately 1 h, then the organic solvent completely disappeared, and the system volume condensed to approximately 5 mL. The condensed system was then quickly moved to an ice-cold environment at 0–2 °C, added to 10 mL of cold water, stirred at the same speed at 1200 rpm for 2 h. The resultant suspension was centrifuged at 20,000 rpm (Avanti J25 centrifuge, JA 25.50 rotor, Beckman Coulter Palo Alto, CA, USA) to remove the supernatant. Finally, the precipitate was resuspended in ultrapure water, refrigerated at −80 °C for 24 h, and lyophilized in a tabletop lyophilizer. The blank SLNs were prepared following the same procedure without the addition of curcumin.

### 4.3. Morphology Determination and Zeta Potential Measurements

The morphology and size of Cur-SLNs were examined by TEM (JEOL, Tokyo, Japan). A diluted Cur-SLNs suspension was dropped onto a copper grid to form a thin liquid film, then negatively stained with 2% (*w*/*v*) sodium phosphotungstate for 10 min, and allowed to air-dry. Then, the images were obtained using TEM at an acceleration voltage of 120 kV.

Zeta potential was estimated at 25 °C on the basis of electrophoretic mobility with a Malvern ZetaSizer Nano ZS (Malvern Instruments, Southborough, UK).

### 4.4. Entrapment Efficiency (EE) and Loading Capacity (LC)

After preparation, the amount of curcumin loaded into SLNs was determined as follows: 2 mL of methanol was added to 5 mg of SLNs, and the mixture was stirred at 37 °C for 2 h. The resulting mixture was centrifuged to separate the undissolved components, and the supernatant containing the drug extracted from SLNs was analyzed using a UV spectrophotometer (Shimadzu, Kyoto, Japan) at 425 nm. The percent EE and LC were calculated by using Equations (1) and (2).

(1) $$\mathrm{EE}\%\;=\;\frac{\mathrm{Total}\;\mathrm{amount}\;\mathrm{of}\;\mathrm{drug}\;\mathrm{added}\;-\;\mathrm{unloaded}\;\mathrm{drug}\;\mathrm{amount}}{\mathrm{Nanoparticle}\;\mathrm{weight}}\times 100\%$$

(2) $$\mathrm{LC}\%\;=\;\frac{\mathrm{Total}\;\mathrm{quantity}\;\mathrm{of}\;\mathrm{drug}\;\mathrm{in}\;\mathrm{SLNs}}{\mathrm{quantity}\;\mathrm{of}\;\mathrm{drug}+\mathrm{quantity}\;\mathrm{of}\;\mathrm{excipients}\;}\times 100\%$$

### 4.5. X-ray Diffraction and Fourier Transform Infrared Spectroscopy (FTIR)

The different crystallographic structures of Cur-SLNs, curcumin, and the SLNs were obtained in the range of 5–50° at ambient temperature with a scanning rate of 0.1° per second. Monochromatic CuΚαradiation (with λ = 1.54060 Å) at 40 kV and 30 mA was used as the X-ray source.

Samples for Fourier transform infrared spectroscopy were vacuum-dried overnight at 60 °C, and recorded using the KBr pellet method on a Nicolet 5700 Fourier transform infrared spectrometer in the range of 4000–400 cm^−1^ at a resolution of 1 cm^−1^.

### 4.6. Cytotoxicity Study and Cellular Uptake Observation

To assess the antitumor efficacy of the Cur-SLNs, SKBR3 cells were treated with various concentrations of free Cur and Cur-SLNs (5, 10, 20, and 40 μM) for 24 h and 48 h. In addition, cells were exposed at the equivalent concentration of blank solid lipid nanoparticles. Afterwards, a sulforhodamine B (SRB) colorimetric assay was performed as we previously described [34]. The cells were fixed in trichloroacetic acid (TCA) for 1 h at 4 °C, and stained with 0.4% SRB dye for 30 min at room temperature. The stained cells were destained with 1% acetic acid, and dissolved in 100 μL of 10 mM Tris buffer (pH 10.5) for 30 min. The optical density of each well was measured at 515 nm on a Bio-Rad 550 ELISA microplate reader. All experiments were performed at least three times.

To analyze the cellular uptake behavior of Cur and Cur-SLNs, SKBR3 cells were seeded in a 6-well plate and grown for 24 h. Then, the medium was changed with fresh solution containing test materials (Cur-SLNs, and Cur at a concentration of 20 μM), the cells were treated for 1 and 6 h, respectively. After that, the cells were washed three times with PBS. Cellular uptake was imaged under the Olympus cell R imaging station to determine the cellular uptake and internalization of nanoparticles.

### 4.7. Wound-Healing Assay

The SKBR3 cells were seeded on 6-well plates at a density of 2 × 10^5^ cells per well, and grown to 90% confluence. Subsequently, the cell monolayer was then scratched with a sterile 200 μL pipette tip to create artificial wounds. After washing and removing any unattached cells, cells were treated with Cur and Cur-SLNs (20 μM) for 0, 8 h, 16 h, 48 h, respectively. Migrated cells were observed under an inverted microscope.

### 4.8. Apoptosis Analysis and Cell Cycle Analysis by Flow Cytometry

Annexin V/propidium iodide (PI) dual-staining method is a sensitive assay for quantitative determination of apoptotic cells [35]. SKBR3 cells were seeded in 6-well plate at a density of 2 × 10^4^ cells/well, and incubated for 24 h for adhesion. Afterwards, old culture media was replaced with fresh culture media, and cells were treated with curcumin or Cur-SLNs for 24 h. Later, the cells were washed twice by PBS, and stained with Annexin V and PI at 37 °C for 30 min. The cells were then analyzed by flow cytometry (BD Bioscience, San Jose, CA, USA) for measuring the proportion of apoptotic cells.

Propidium iodide (PI) staining of cells and subsequent analysis by flow cytometry was carried out to analyze the cell cycles. After overnight plating in 6-well per well, cells were incubated with 20 μM curcumin, Cur-SLNs for 24 h. The cells were harvested with trypsin 24 h after treatment, washed twice with PBS, and 1.0 × 10^6^ cells were suspended in the binding buffer of the Annexin V kit. Then, cells were resuspended in precooled 70% ethanol, and fixed by storage on ice for at least 2 h. After washing with ice-cold PBS, to prevent inadvertent staining of dsRNA, the cells were treated with 50 μL RNaseA at 37 °C for 30 min. Ultimately, the cells were washed with PBS and stained with propidium iodide for 30 min at room temperature. Next, the flow cytometry analysis was carried out for 10,000 events per sample through FL2-A band-pass filter (PI) using flow cytometry.

### 4.9. Intracellular ROS Detection

SKBR3 cells were plated in 6-well plates at 37 °C for 24 h. Then, the medium was replaced with fresh medium, and treated with free Cur and Cur-SLNs (20 μM of free Cur equivalent) for 24 h. After treatment, the cells were washed with PBS and harvested. Then, 150 μL of 10 μM 2′,7′-dichlorofluorescin diacetate was added to the cell suspension and incubated for 1 h at room temperature in the dark. Exactly 10,000 cells were analyzed with analytical flow cytometry instrument (BD Bioscience, San Jose, CA, USA).

### 4.10. Western Blot Analysis

SKBR3 cells treated with Cur or Cur-SLNs, and then harvested and lysed in RIPA lysis buffer (Beyotime, Shanghai, China) with freshly added PMSF (Beyotime) for 30 min on ice. Samples were centrifuged at 12,000 rpm for 10 min, the supernatant was subsequently collected and stored at −80 °C for preservation, and the protein concentration was measured using Bio-Rad Protein Assay kit (Bio-Rad, Hercules, CA, USA) The samples were subjected to 10% SDS-PAGE following heat denaturation at 95 °C for 5 min. Subsequently, the target proteins in the gel were transferred onto polyvinylidene difluoride (PVDF) membranes (Bio-Rad Laboratories, Inc., Hercules, CA, USA) and the membranes were blocked with 5% BSA for 90 min at room temperature. The membranes were incubated with the following primary antibodies: *Bcl-2* (12789-1-AP; dilution 1:1000), *Bax* (60267-1-lg, dilution 1:5000), *CDK4* (11026-1-AP, dilution 1:2000) and Cyclin D1 (60186-1-lg, dilution 1:5000) at 4 °C overnight. Quantification of protein bands was performed using the ImageJ software.

### 4.11. Statistical Analysis

All data were displayed as a mean ± standard error. A one-way analysis of variance (ANOVA) followed by Dunnett’s analysis was performed, and values of *p* < 0.05 or 0.01 were considered significant.

## 5. Conclusions

In summary, we have successfully incorporated curcumin into solid lipid nanoparticles to improve its biological efficacy against SKBR3 cells in vitro. The optimized formulation had a mean particle size of about 30 nm, and negative zeta potential. Consequently, the incorporation of curcumin in SLNs remarkably increased the cell death and induced apoptotic compared to free curcumin. Moreover, we observed that Cur-SLNs could inhibit cell migration. Taken together, the results indicated that the SLNs could be better a good carrier for curcumin.
